# Resveratrol Protects Photoreceptors in Mouse Models of Retinal Degeneration

**DOI:** 10.3390/antiox14020154

**Published:** 2025-01-28

**Authors:** Shujuan Li, Hongwei Ma, Xi-Qin Ding

**Affiliations:** Department of Cell Biology, University of Oklahoma Health Sciences Center, Oklahoma City, OK 73104, USA; shujuan-li@ouhsc.edu (S.L.); hongwei-ma@ouhsc.edu (H.M.)

**Keywords:** resveratrol, thyroid hormone, photoreceptor, retinal degeneration, mice

## Abstract

Photoreceptor/retinal degeneration is the major cause of blindness. Induced and inherited mouse models of retinal degeneration are valuable tools for investigating disease mechanisms and developing therapeutic interventions. This study investigated the potential of the antioxidant resveratrol to relieve photoreceptor degeneration using mouse models. Clinical studies have shown a potential association between thyroid hormone (TH) signaling and age-related retinal degeneration. Excessive TH signaling induces oxidative stress/damage and photoreceptor death in mice. C57BL/6 (rod-dominant) and *Nrl^−/−^* (cone-dominant) mice at postnatal day 30 (P30) received triiodothyronine (T3) via drinking water (20 µg/mL) with or without concomitant treatment with resveratrol via drinking water (120 µg/mL) for 30 days, followed by evaluation of photoreceptor degeneration, oxidative damage, and retinal stress responses. In experiments using Leber congenital amaurosis model mice, mother *Rpe65^−/−^* and *Rpe65^−/−^*/*Nrl^−/−^* mice received resveratrol via drinking water (120 µg/mL) for 20 days and 10–13 days, respectively, beginning on the day when the pups were at P5, and pups were then evaluated for cone degeneration. Treatment with resveratrol significantly diminished the photoreceptor degeneration induced by T3 and preserved photoreceptors in *Rpe65*-deficient mice, manifested as preserved retinal morphology/outer nuclear layer thickness, increased cone density, reduced photoreceptor oxidative stress/damage and apoptosis, reduced upregulation of genes involved in cell death/inflammatory responses, and reduced macroglial cell activation. These findings demonstrate the role of oxidative stress in photoreceptor degeneration, associated with TH signaling and *Rpe65* deficiency, and support the therapeutic potential of resveratrol/antioxidants in the management of retinal degeneration.

## 1. Introduction

Rod and cone photoreceptors are specialized sensory neurons in the retina responsible for capturing visual information. Rods are responsible for dim light vision, whereas cones are responsible for bright light vision, color vision, and visual acuity. Progressive photoreceptor degeneration occurs in inherited retinal diseases, such as Leber congenital amaurosis (LCA), retinitis pigmentosa, and cone–rod dystrophies, and age-related retinal diseases, such as age-related macular degeneration and diabetic retinopathy. This degeneration ultimately leads to vision impairment and blindness. The highly heterogeneous nature of these diseases is a challenge for the development of therapeutic strategies, and there are currently no cures for retinal degeneration. Nevertheless, mouse models of induced or inherited retinal degeneration remain valuable tools for studying disease mechanisms and exploring therapeutic strategies.

Thyroid hormone (TH) signaling is known to regulate cell proliferation, differentiation, and metabolic homeostasis. Clinical studies have shown that abnormalities in TH signaling are linked to neurodegenerative conditions/dementia in the elderly, including Alzheimer’s disease [1,2,3] and age-related retinal disease [4,5,6,7,8,9,10,11,12,13]. Optical coherence tomography evaluations have further demonstrated macular thinning in patients with thyroid-associated ophthalmopathy [14,15]. In the retina, TH signaling plays a significant role in photoreceptor cell death and survival. Studies in mice have shown that excessive TH signaling, whether through triiodothyronine (T3) treatment or deletion of the T3-degrading enzyme DIO3, leads to retinal oxidative stress/damage and photoreceptor degeneration [16,17,18]. Moreover, transcriptomic analysis revealed that excessive TH signaling impairs mitochondrial bioenergetics and induces cellular stress in mouse photoreceptors [19]. In contrast, suppression of TH signaling reduced photoreceptor degeneration in mouse models of retinal degeneration, including the LCA model of *Rpe65*-deficient mice [16,20,21,22,23]. LCA is a severe autosomal recessive blinding disease that appears at birth or in the first few months of life and affects 2–3 out of 100,000 births [24]. The disease accounts for blindness in over 20% of children attending schools for the blind. There are about twenty-four genes that are associated with LCA. Among these, mutations in the *RPE65* gene, which encodes the retinal pigment epithelium-specific isomerohydrolase RPE65, account for approximately 16% of all LCA cases (https://sph.uth.edu/retnet/).

Resveratrol is a natural polyphenolic compound abundant in grape juice, cereals, peanuts, pine, and berries [25,26] and possesses potent antioxidant properties [27,28]. This study aimed to determine the beneficial effects of resveratrol in mice of both induced and inherited retinal degeneration. Specifically, we examined the effects of resveratrol in mice treated with T3 and in an LCA model of *Rpe65*-deficient mice. We showed that treatment with resveratrol significantly mitigated T3-induced photoreceptor degeneration and preserved photoreceptors in *Rpe65*-deficient mice. This study demonstrated the role of oxidative stress/damage in retinal degeneration in the examined models, and the findings support the potential therapeutic application of resveratrol/antioxidants in the management of retinal degeneration.

## 2. Materials and Methods

### 2.1. Mice, Antibodies, and Reagents

The C57BL/6J mouse line was obtained from the Jackson Laboratory. The *Nrl*^−/−^ mouse line [29] was provided by Dr. Anand Swaroop (Neurobiology Neurodegeneration & Repair Laboratory, NEI), and the *Rpe65^−/−^* mouse line [30] was provided by Dr. T. Michael Redmond (Laboratory of Retinal Cell and Molecular Biology, NEI). The *Rpe65^−/−^/Nrl^−/−^* line was generated by cross-breeding [21,30,31]. All mice were maintained under cyclic-light (12 h light–dark) conditions. Cage illumination was ~7 foot candles during the light cycle. All animal maintenance and experiments were approved by the local Institutional Animal Care and Use Committee (University of Oklahoma Health Sciences Center, protocol number: 23-038-EH) and conformed to the guidelines on the Care and Use of Animals of the Society for Neuroscience and the Association for Research in Vision and Ophthalmology (Rockville, MD, USA). Mice of both genders were used. The antibodies and reagents used in the experiments were listed in Table 1.

### 2.2. Treatment with T3 and Resveratrol

T3 for drinking water administration was prepared as described previously [32]. Ten milligrams of T3 (Sigma-Aldrich, St. Louis, MO, USA, Catalog#: T2877) was dissolved in 1.0 mL of 1.0 N NaOH, followed by dilution with tap water for a final working concentration of 20 µg/mL. For resveratrol drinking water preparation, 12 mg of resveratrol (Sigma-Aldrich, Catalog#: R5010) was dissolved in 200 μL of absolute ethanol, and then diluted in 100 mL tap water to a final working concentration of 120 µg/mL [33]. Water bottles were covered with aluminum foil to avoid light exposure, and the drinking water was replaced weekly. Postnatal day 30 (P30) C57BL/6 and *Nrl^−/−^* mice received T3 with or without concomitant treatment of resveratrol for 30 days. Control/untreated mice received vehicle for resveratrol (0.2% ethanol in drinking water). At the end of the treatments, the eyes/retinas of these mice were collected for analysis of photoreceptor viability/cell death and cellular stress responses. Due to the early onset and rapid degeneration in *Rpe65*-deficient mice, antioxidant treatment was initiated at a young age. Mother *Rpe65^−/−^* and *Rpe65^−/−^*/*Nrl^−/−^* mice received resveratrol via drinking water (120 µg/mL) or vehicle for 20 days and 10–13 days, respectively, beginning on the day when the pups were at P5. At the end of the treatments, eyes/retinas of pups were collected for analysis of photoreceptor viability/cell death and cellular stress responses.

### 2.3. Eye Preparation, Immunofluorescence Labeling, Confocal Microscopy, and Morphometric Analysis

Mouse retinal whole mounts or cross sections were prepared for immunofluorescence labeling and morphometric analysis as described previously [16,17,21]. For retinal whole-mount preparations, eyes were enucleated, marked at the superior (dorsal) pole with a green dye for orientation, then fixed in 4% paraformaldehyde (PFA) (Polysciences, Inc., Warrington, PA, USA) for 60 min at room temperature, followed by removal of the cornea and lens. The eyes were then fixed in 4% PFA for 4–6 h at room temperature, the retinas were isolated, and the superior portion was marked for orientation with a small cut. For retinal cross sections, the superior portion of the cornea was marked with green dye for dorsal orientation, and mouse eyes were enucleated and fixed in Prefer (Anatech Ltd., Battle Creek, MI, USA) for 25 min at room temperature. Fixed eyes were stored in 70% ethanol until processing for sections. Paraffin sections (5 µm thickness) passing vertically through the retina (along the vertical meridian passing through the optic nerve head) were prepared using a Leica microtome (Leica Biosystems, Nussloch, Germany).

Immunofluorescence labeling was performed as described previously [16,17,21]. In brief, retinal whole mounts were blocked with Hanks’ balanced salt solution containing 10% bovine serum albumin (BSA) (Sigma-Aldrich) and 0.5% Triton X-100 (Bio-Rad, Hercules, CA, USA) for 1 h at room temperature. Peanut-agglutinin (PNA) immunohistochemistry was achieved by biotinylated PNA labeling overnight at 4 °C and then streptavidin-Cy3 labeling at room temperature for 1 h. For immunofluorescence labeling on retinal sections, after deparaffinization and rehydration steps, antigen retrieval was conducted in 25 mM Tris (PH = 8.5) and 1 mM EDTA buffer [34] for 30 min in a 70.5 °C water bath. Primary antibody incubation was performed overnight at 4 °C, followed by incubation with Alexa Fluor^TM^ 555 goat anti-rabbit secondary antibody at room temperature for 2 h. Table 1 shows the dilutions of the antibodies used. Immunofluorescent signals were imaged using an Olympus FV1000 confocal laser scanning microscope (Olympus, Melville, NY, USA) with FluoView imaging software (Olympus). Evaluation of cone density on retinal whole mounts, evaluation of GFAP fluorescence density on retinal sections, and quantitative analysis of the number of *p*-γH2AX-positive cells were performed as described previously [17,35]. For retinal morphometric analysis, retinal cross sections stained with hematoxylin and eosin (H&E) were used to evaluate outer nuclear layer (ONL) thickness/rod survival, as described previously [17,36,37].

### 2.4. TUNEL Assay

Terminal deoxynucleotidyltransferase dUTP nick-end labeling (TUNEL) was performed on paraffin-embedded retinal sections, using an In Situ Cell-Death Fluorescein-Detection kit (Sigma-Aldrich, Catalog#: 11684795910), as described previously [38]. Immunofluorescence signals were imaged using an Olympus FV1000 confocal laser-scanning microscope. TUNEL-positive cells in the ONL passing through the optic nerve were counted and averaged from at least three sections per eye, from 3 to 11 mice per condition.

### 2.5. Retinal Protein Preparation, SDS-PAGE, and Western Blot Analysis

Retinal protein preparation, SDS-PAGE, and Western blot analysis were prepared as described previously [21,23,39]. Retinal tissues were homogenized in homogenization buffer (0.32 M sucrose, 20 mM 4-(2-hydroxyethyl)-1-piperazineethanesulfonic acid (HEPES), pH 7.4, and 3 mM EDTA containing protease and phosphatase inhibitors (Roche Applied Science, Indianapolis, IN, USA, Catalog #: 04693159001 and Catalog #: 04906837001, respectively)). The homogenates were centrifuged at 3000 rpm for 10 min at 4 °C, and the resulting supernatant was then centrifuged at 13,000 rpm for 35 min at 4 °C to separate cytosolic (supernatant) and membrane (pellet) fractions. The cytosolic fractions were used, and protein concentrations were measured with a protein assay kit from Bio-Rad Laboratories. Retinal protein samples were subjected to SDS-PAGE and transferred to PVDF membranes, which were subsequently blocked in 5% BSA for 1 h at room temperature. Immunoblots were incubated with primary antibody overnight at 4 °C. Table 1 shows dilutions of the antibodies used. After washing in Tris-buffered saline with 0.1% Tween 20, immunoblots were incubated with HRP-conjugated anti-rabbit or anti-mouse secondary antibody for 1 h at room temperature. SuperSignal^®^ West Dura Extended Duration chemiluminescent substrate (Thermo Fisher Scientific, Carlsbad, CA, USA) was used to detect binding of the primary antibodies to their cognate antigens. Li-Cor Odyssey CLx Imager and Li-Cor software (Li-Cor Biosciences, Lincoln, NE, USA) were used for detection and densitometric analysis.

### 2.6. RNA Isolation and Quantitative Real-Time PCR

Total RNA preparation and reverse transcription were performed as described previously [36,37]. Briefly, retinas were lysed and RNA isolated using the PureLink^TM^ RNA kit (Thermo Fisher Scientific) as per the manufacturer’s instructions. cDNA was prepared using iScript Reverse Transcription Supermix (Bio-Rad), and the obtained cDNA was amplified using iTaq Universal SYBR^®^ Green Supermix (Bio-Rad). The primer sets are listed in Table 2. The gene encoding the mouse hypoxanthine guanine phosphoribosyl transferase 1 (*Hprt1*) was included as an internal control. The quantitative real-time PCR (qRT-PCR) assays were performed using a CFX connected Real-Time PCR Detection System (iCycler, Bio-Rad Laboratories, Hercules, CA, USA). All assessed genes were run in triplicate, and the relative gene expression was calculated based on the ΔΔCt method with conditions normalized to *Hprt1*.

### 2.7. Statistical Analysis

Results are expressed as means ± *SEM* of the number of mice or the number of assays. One-way ANOVA was used to test for significant differences within sets of data, and the unpaired Student’s *t* test was used to assess significance between two groups of data. Differences were considered statistically significant when *p* < 0.05. Data were analyzed and graphed using GraphPad Prism^®^ software (GraphPad Software, San Diego, CA, USA).

## 3. Results

### 3.1. Treatment with Resveratrol Mitigated T3-Induced Photoreceptor Cell Loss/Degeneration in C57BL/6 Mice

**Figure 1:**
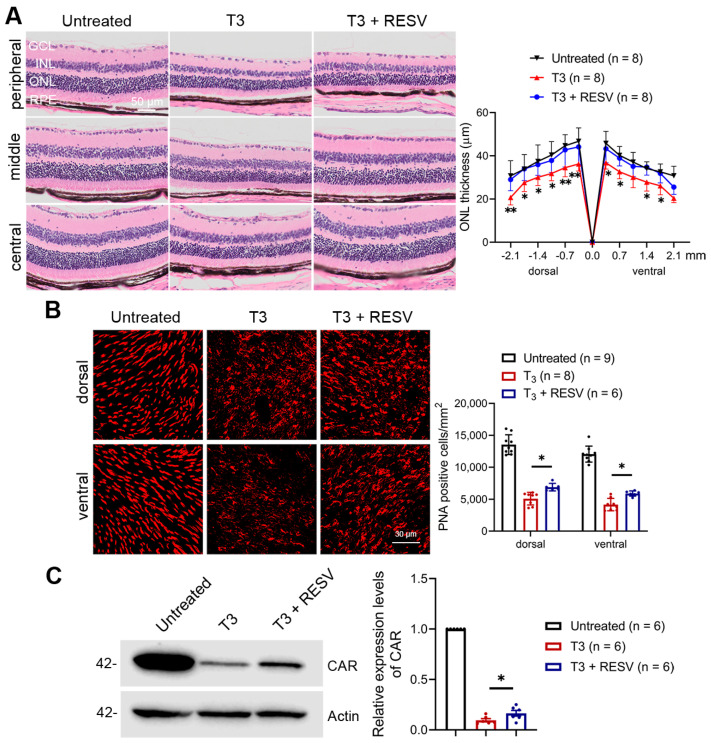
Treatment with resveratrol reduced T3-induced photoreceptor cell loss/degeneration in C57BL/6 mice. (**A**) Shown are representative light microscopic images of H&E-stained retinal sections and corresponding quantitative analysis of ONL thickness. (**B**) Shown are representative confocal images of PNA labeling on retinal whole mounts and corresponding quantitative analysis. (**C**) Shown are representative immunoblotting images with corresponding quantitative analysis for cone arrestin (CAR). GCL, ganglion cell layer; INL, inner nuclear layer; ONL, outer nuclear layer; RPE, retinal pigment epithelial; RESV, resveratrol. Data are represented as means ± *SEM* for 6–9 mice per group (**A**,**B**) and of 6 assays using retinas prepared from 3–4 mice per group (**C**) (* *p* < 0.05, ** *p* < 0.01).

We first examined whether treatment with resveratrol will preserve photoreceptors. P30 C57BL/6 mice received T3 treatment (20 µg/mL in drinking water) without or with concomitant treatment of resveratrol (120 µg/mL in drinking water) for 30 days, followed by evaluation of rod and cone survival/degeneration. Retinal integrity/rod survival was evaluated by morphometric analysis on H&E-stained retinal sections. The analysis showed photoreceptor layer damage after T3 treatment, including reduced ONL thickness and a shortened outer segment (Figure 1A). Concomitant treatment with resveratrol significantly preserved retinal morphology and reduced photoreceptor/rod cell loss. The ONL thickness in the central retina was reduced by about 25% in mice after T3 treatment, and treatment with resveratrol prevented the cell loss and preserved ONL thickness (Figure 1A). Cone survival/degeneration was evaluated by PNA labeling on retinal whole mounts. Treatment with T3 reduced cone density by about 70% compared with untreated controls. Concomitant treatment with resveratrol significantly increased cone density compared with mice receiving T3 only (Figure 1B). We also evaluated the expression levels of the cone-specific protein cone arrestin (CAR) in the retina by immunoblotting. The expression level of CAR was significantly reduced after T3 treatment, and concomitant treatment with resveratrol increased the expression of CAR compared with mice receiving T3 only (Figure 1C). These data show that treatment with resveratrol mitigated T3-induced photoreceptor cell loss/degeneration.

### 3.2. Treatment with Resveratrol Reduced T3-Induced Photoreceptor Apoptosis in C57BL/6 and Nrl^−/−^ Mice

**Figure 2:**
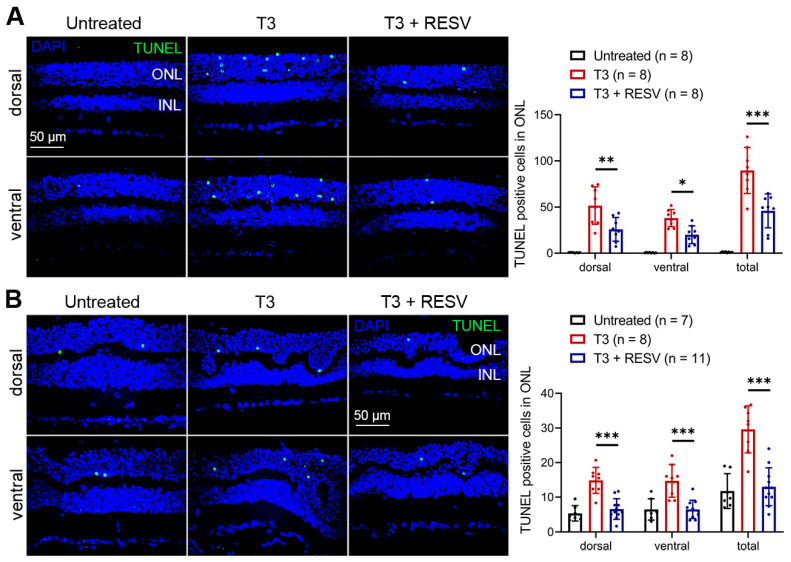
Treatment with resveratrol reduced T3-induced photoreceptor apoptosis in C57BL/6 and *Nrl*^−/−^ mice. Shown are representative confocal images of TUNEL labeling on retinal sections prepared from C57BL/6 mice (**A**) and *Nrl*^−/−^ mice (**B**) and corresponding quantitative analysis of TUNEL-positive cells. ONL, outer nuclear layer; INL, inner nuclear layer; RESV, resveratrol. Data are represented as means ± *SEM* for 7–11 mice per group (* *p* < 0.05, ** *p* < 0.01, *** *p* < 0.001).

We next examined the effects of resveratrol on T3-induced photoreceptor apoptosis. Both C57BL/6 and *Nrl*^−/−^ mice were used. Mice lacking NRL, a rod-specific neural retina leucine zipper transcriptional factor, have a cone-dominant retina [29]. The use of *Nrl*^−/−^ mice facilitates the evaluations of cones owing to the sparse number of cones in a mouse retina (cones represent only 3–5% of the total photoreceptor population in a murine retina). The detrimental effects of excessive TH signaling have also been shown in *Nrl*^−/−^ mice [17]. P30 mice received T3 treatment (20 µg/mL in drinking water) without or with concomitant treatment of resveratrol (120 µg/mL in drinking water) for 30 days, followed by evaluation of photoreceptor apoptosis using TUNEL on retinal cross sections. The numbers of TUNEL-positive cells in T3-treated C57BL/6 and *Nrl*^−/−^ mice were significantly increased compared with their respective untreated controls (Figure 2). Concomitant treatment with resveratrol greatly reduced T3-induced photoreceptor apoptosis (Figure 2).

### 3.3. Treatment with Resveratrol Reduced T3-Induced Oxidative Stress/Damage in C57BL/6 and Nrl^−/−^ Mice

**Figure 3:**
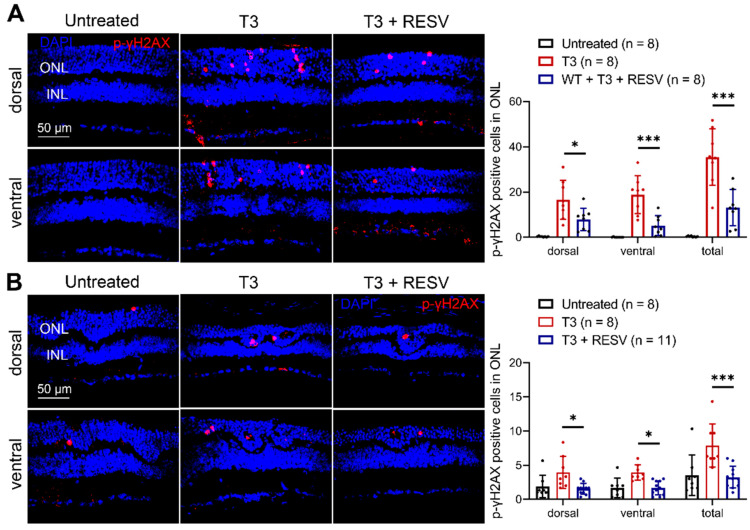
Treatment with resveratrol reduced T3-induced oxidative stress/damage in the retinas of C57BL/6 and *Nrl*^−/−^ mice. Shown are representative confocal images of *p*-γH2AX immunolabeling on retinal sections and corresponding quantitative analysis in C57BL/6 mice (**A**) and *Nrl*^−/−^ mice (**B**). ONL, outer nuclear layer; INL, inner nuclear layer; RESV, resveratrol. Data are represented as means ± *SEM* of 8–11 mice per group (* *p* < 0.05, *** *p* < 0.001).

We further evaluated the effects of resveratrol on T3-induced oxidative stress/damage in the retina. P30 C57BL/6 and *Nrl*^−/−^ mice received T3 treatment (20 µg/mL in drinking water) without or with concomitant treatment of resveratrol (120 µg/mL in drinking water) for 30 days and were then analyzed for oxidative damage using labeling of the DNA damage marker *p*-γH2AX on retinal cross sections. Treatment with T3 greatly increased the number of *p*-γH2AX-positive cells in C57BL/6 and *Nrl*^−/−^ mice compared with their respective untreated controls (Figure 3). Concomitant treatment of resveratrol greatly diminished this increase in C57BL/6 mice (Figure 3A) and completely abolished the increase in *Nrl*^−/−^ mice (Figure 3B) compared with mice that were treated with T3 only. These data show that treatment with resveratrol reduced T3-induced oxidative stress/damage in the retina.

### 3.4. Treatment with Resveratrol Inhibited T3-Induced Macroglial Cell Activation and Upregulation of Genes Involved in Cell Stress/Death and Inflammatory Responses in C57BL/6 and Nrl^−/−^ Mice

**Figure 4:**
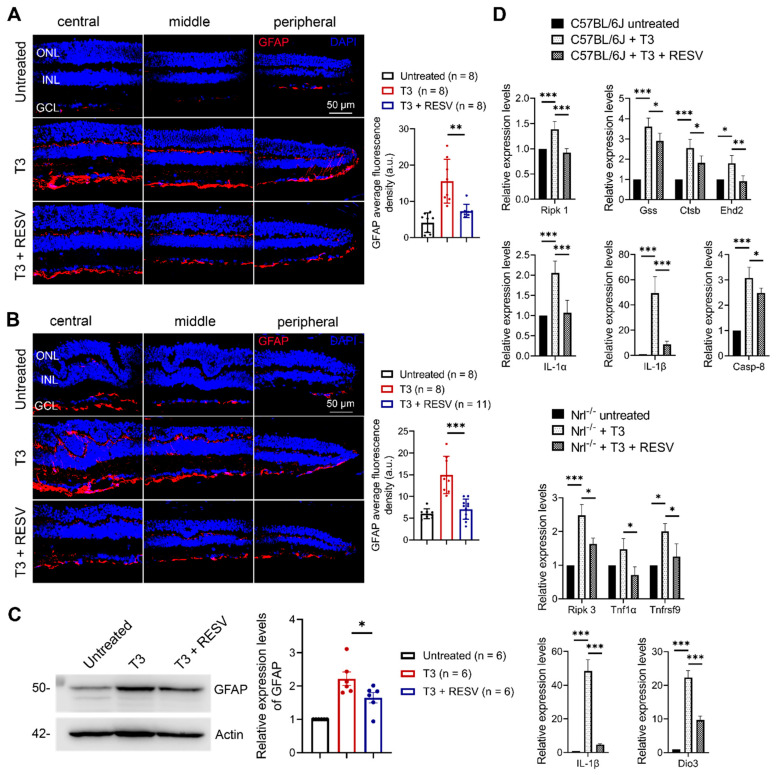
Treatment with resveratrol inhibited T3-induced macroglial cell activation in C57BL/6 and *Nrl^−/−^* mice. (**A**,**B**) Shown are representative confocal images of GFAP labeling on retinal sections prepared from C57BL/6 mice (**A**) and *Nrl^−/−^* mice (**B**) and corresponding quantitative analysis of fluorescence density. (**C**) Shown are representative images of GFAP immunoblotting using retinas prepared from C57BL/6 mice and corresponding quantitative analysis. (**D**) Shown are qRT-PCR results for expression levels of genes involved in oxidative stress, cell death, and inflammatory response. ONL, outer nuclear layer; INL, inner nuclear layer; GCL, ganglion cell layer; RESV, resveratrol. Data are represented as means ± *SEM* for 8–11 mice per group (**A**,**B**) and of 6 assays using retinas prepared from 3–4 mice per group (**C**,**D**) (* *p* < 0.05, ** *p* < 0.01, *** *p* < 0.001).

Macroglial cells (Müller cells and astrocytes) activate in response to retinal stress insults via increased expression of glial fibrillary acidic protein (GFAP) [40], and treatment with T3 increases expression of GFAP [17]. In this work, we examined the effect of resveratrol on T3-induced macroglial cell activation. P30 C57BL/6 and *Nrl*^−/−^ mice received T3 treatment (20 µg/mL in drinking water) without or with concomitant treatment with resveratrol (120 µg/mL in drinking water) for 30 days and were then analyzed for macroglial cell activation by GFAP labeling on retinal cross sections and by immunoblotting. The fluorescence density of GFAP was increased by about 3-fold in mice after T3 treatment (Figure 4A,B). Concomitant treatment with resveratrol greatly diminished the GFAP signal in C57BL/6 mice (Figure 4A) and completely abolished the increase in *Nrl^−/−^* mice (Figure 4B) compared with mice treated with T3 only. Consistent with the findings in GFAP labeling, immunoblotting of GFAP using retinal protein extracts showed that the level of GFAP was increased by 1-fold in the T3-treated mice compared with untreated controls, and treatment with resveratrol significantly diminished this elevation (Figure 4C). We also examined the effects of resveratrol on T3-induced upregulation of the genes involved in oxidative stress, cell death signaling, and inflammation. Several genes regulating oxidative stress (*Gss*, *Ctsb*, *Ehd2*), cell death signaling (*Tnf1α*, *Tnfrsf9*, *Ripk1*, *Ripk3*, *Casp-8*), and inflammation (*Il-1α, Il-1β*) were significantly upregulated in the retinas of mice after T3 treatment, whereas treatment with resveratrol inhibited these elevations (Figure 4D).

### 3.5. Treatment with Resveratrol Preserved Cones in Rpe65^−/−^ and Rpe65^−/−^/Nrl^−/−^ Mice

**Figure 5:**
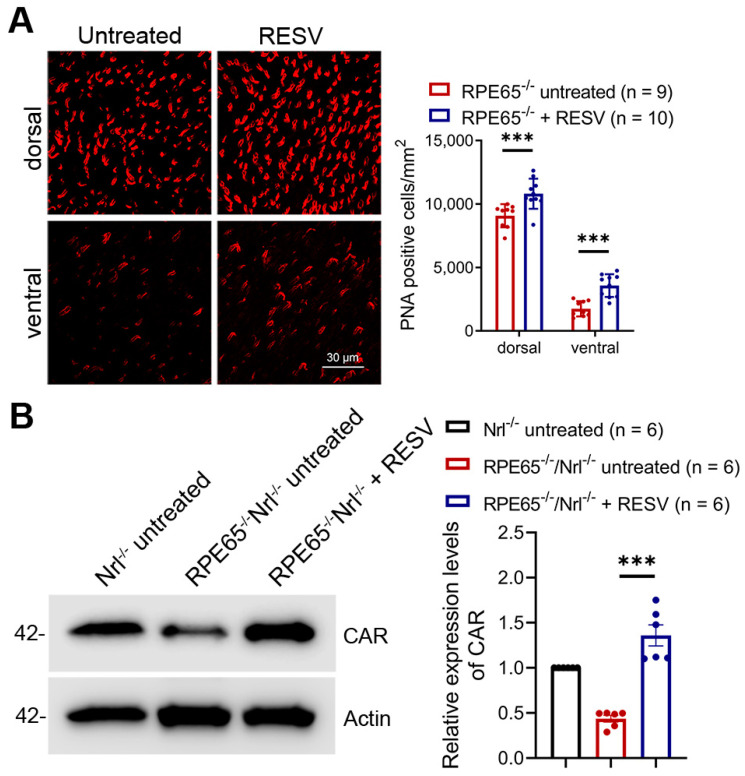
Treatment with resveratrol preserved cones in *Rpe65^−/−^* mice and *Rpe65^−/−^*/*Nrl^−/−^* mice. (**A**) Shown are representative confocal images of PNA labeling on retinal whole mounts prepared from *Rpe65^−/−^* mice and corresponding quantitative analysis. (**B**) Shown are the results of immunoblotting with corresponding quantitative analysis for CAR, using retinas prepared from *Rpe65^−/−^*/*Nrl^−/−^* mice. RESV, resveratrol; CAR, cone arrestin. Data are represented as means ± *SEM* for 9–10 mice per group (**A**) and of 6 assays using retinas prepared from 4–5 mice per group (**B**) (*** *p* < 0.001).

*Rpe65^−/−^* and *Rpe65^−/−^/Nrl^−/−^* mice display early-onset and rapid cone degeneration and have been commonly used as models for studies of LCA [30,31,41,42]. In this work, we examined the effects of resveratrol. Mother *Rpe65^−/−^* and *Rpe65^−/−^*/*Nrl^−/−^* mice received resveratrol (120 µg/mL in drinking water) or vehicle for 20 days and 10–13 days, respectively, beginning on the day when the pups were at P5, and pups were then evaluated for cone degeneration. Cone density evaluation on *Rpe65^−/−^* mice by PNA labeling showed a degeneration pattern similar to that reported previously [42,43], i.e., the ventral retina shows early-onset, fast cone degeneration (about 10% of the wild-type level remained at P30), whereas the peripheral dorsal retina was relatively less degenerated (about 50% of the wild-type level remained at P30). Treatment with resveratrol effectively preserved cones in both dorsal and ventral areas. Cone density was increased by about 20–30% in the dorsal area and about 2-fold in the ventral area, compared with untreated controls (Figure 5A). Immunoblotting analysis was performed using retinal protein extracts prepared from *Rpe65^−/−^*/*Nrl^−/−^* mice and showed that the level of CAR was greatly increased in resveratrol-treated mice compared with untreated controls (Figure 5B).

### 3.6. Treatment with Resveratrol Reduced Photoreceptor Apoptosis in Rpe65^−/−^ and Rpe65^−/−^/Nrl^−/−^ Mice

**Figure 6:**
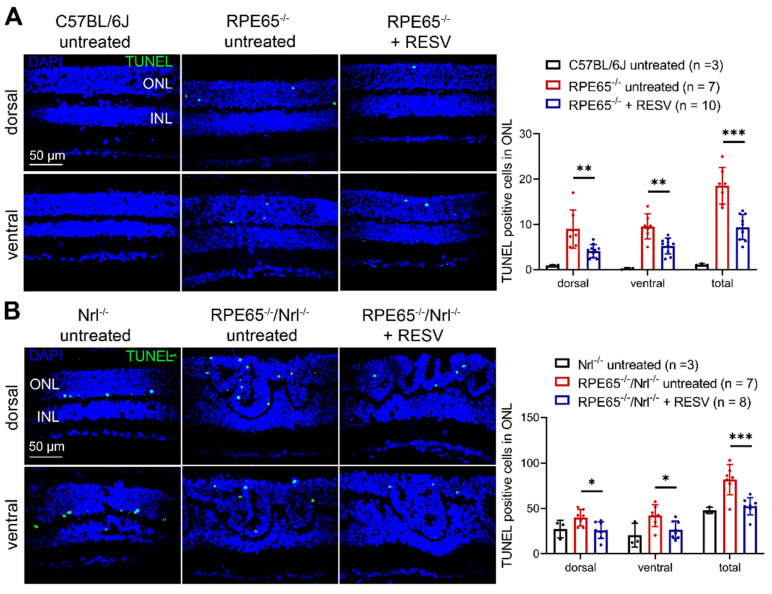
Treatment with resveratrol reduced photoreceptor apoptosis in *Rpe65^−/−^* mice and *Rpe65^−/−^*/*Nrl^−/−^* mice. Shown are representative confocal images of TUNEL labeling on retinal sections prepared from *Rpe65^−/−^* mice (**A**) and *Rpe65^−/−^*/*Nrl^−/−^* mice (**B**) and corresponding quantitative analysis. ONL, outer nuclear layer; INL, inner nuclear layer; RESV, resveratrol. Data are represented as means ± *SEM* of 3–10 mice per group (* *p* < 0.05, ** *p* < 0.01, *** *p* < 0.001).

We then examined the effects of resveratrol on photoreceptor apoptosis in *Rpe65*-deficient mice. Mother *Rpe65^−/−^* and *Rpe65^−/−^*/*Nrl^−/−^* mice received resveratrol (120 µg/mL in drinking) or vehicle for 20 days and 10–13 days, respectively, beginning on the day when the pups were at P5, and pups were then evaluated for photoreceptor apoptosis. The number of TUNEL-positive cells in *Rpe65^−/−^* and *Rpe65^−/−^*/*Nrl^−/−^* mice was significantly higher than that in their respective controls (Figure 6). Treatment with resveratrol reduced this number by about 50% in *Rpe65^−/−^* mice (Figure 6A) and about 45% in *Rpe65^−/−^/Nrl^−/−^* mice (Figure 6B) compared with their respective untreated controls.

### 3.7. Treatment with Resveratrol Inhibited Macroglial Cell Activation and Upregulation of Genes Involved in Cell Stress/Death and Inflammatory Responses in Rpe65^−/−^ and Rpe65^−/−^/Nrl^−/−^ Mice

**Figure 7:**
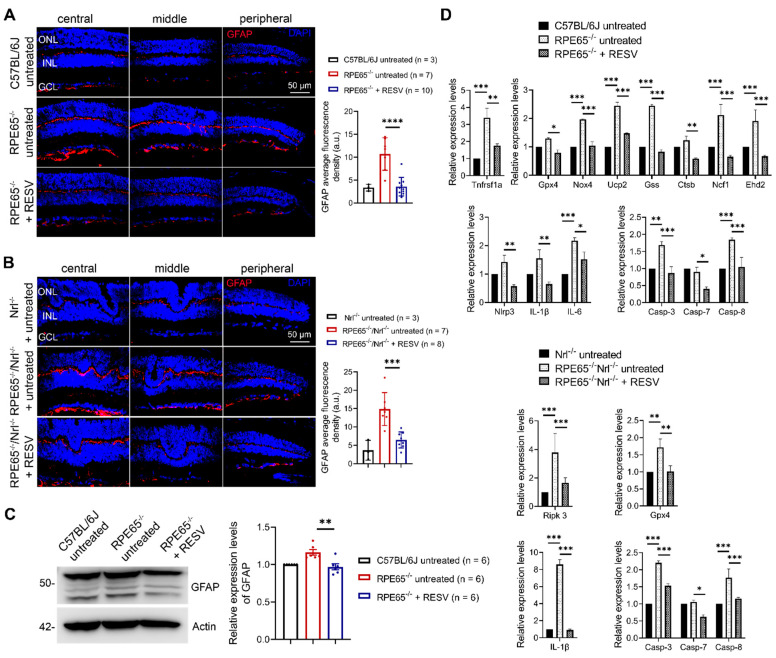
Treatment with resveratrol inhibited macroglial cell activation in *Rpe65^−/−^* mice and *Rpe65^−/−^*/*Nrl^−/−^* mice. (**A**,**B**) Shown are representative confocal images of GFAP labeling on retinal sections prepared from *Rpe65^−/−^* mice (**A**) and *Rpe65^−/−^*/*Nrl^−/−^* mice (**B**) and corresponding quantitative analysis of fluorescence density. (**C**) Shown are the results of immunoblotting of GFAP using retinas prepared from *Rpe65^−/−^* mice with corresponding quantitative analysis. (**D**) Shown are the qRT-PCR results for expression levels of genes involved in oxidative stress, cell death, and the inflammatory response. ONL, outer nuclear layer; INL, inner nuclear layer; GCL, ganglion cell layer; RESV, resveratrol; Data are represented as means ± *SEM* for 3–10 mice per group (**A**,**B**) and of 6 assays using retinas prepared from 3–7 mice per group (**C**,**D**) * *p* < 0.05, ** *p* < 0.01, *** *p* < 0.001).

We further examined the effects of resveratrol on the retinal stress response/macroglial cell activation in *Rpe65*-deficient mice. Mother *Rpe65^−/−^* and *Rpe65^−/−^*/*Nrl^−/−^* mice received resveratrol (120 µg/mL in drinking water) or vehicle for 20 days and 10–13 days, respectively, beginning on the day when the pups were at P5, and pups were then evaluated for GFAP expression by immunofluorescence labeling and immunoblotting. The fluorescence density of GFAP in *Rpe65^−/−^* and *Rpe65^−/−^*/*Nrl^−/−^* mice was greatly increased compared with their respective controls (Figure 7A,B). Treatment with resveratrol completely abolished the elevation in *Rpe65^−/−^* mice (Figure 7A) and greatly diminished the elevation in *Rpe65^−/−^/Nrl^−/−^* mice (Figure 7B) compared with their respective untreated controls. Consistent with the findings in GFAP labeling, immunoblotting of GFAP using retinal protein extracts showed that the level of GFAP was significantly increased in *Rpe65^−/−^* mice compared with wild-type (C57BL/6) controls, and treatment with resveratrol abolished this elevation (Figure 7C). We also examined the effects of resveratrol on the expression of genes involved in cell death, oxidative stress, and inflammation. Several genes regulating oxidative stress (*Nox4*, *Ucp2*, *Gss*, *Ctsb*, *Ehd2*), cell death (*Tnfrsf1α*, *Ripk3*, *Casp-3, Casp-8*), and inflammation (*Nirp3, Il-6, Il-1β*) were significantly elevated in *Rpe65^−/−^* and *Rpe65^−/−^*/*Nrl^−/−^* mice, whereas treatment with resveratrol inhibited these elevations (Figure 7D).

## 4. Discussion

### 4.1. Resveratrol Preserves Photoreceptors Against TH-Induced Degeneration

It has been well documented that excessive TH signaling induces rod and cone degeneration and cell death, accompanied by oxidative stress/damage [16,17,18,19]. In this work, we evaluated the effects of resveratrol to address the role of oxidative stress in TH-signaling-induced photoreceptor degeneration and explore its potential therapeutic benefits. Treatment with resveratrol led to rod and cone protection against T3-induced damage, as shown by increased ONL thickness, cone density, and expression of cone arrestin, a reduced number of TUNEL-positive cells and oxidative stress/damage, and inhibition of the upregulation of genes involved in the cellular stress response and death process. As TUNEL-positive cells were mainly detected in the ONL, where photoreceptors are localized, it is likely that the dying cells are photoreceptors. However, TUNEL and immunofluorescence double-labeling with a photoreceptor-specific marker would provide stronger evidence for photoreceptor death. These results support the role of oxidative stress in T3-induced photoreceptor degeneration. Similar results have been reported previously, showing that administration of N-acetyl cysteine (an antioxidant) reduces T3-induced cone degeneration and retinal stress [17]. Clinical studies have shown that abnormalities in TH signaling are linked to age-related retinal degeneration [4,5,6,7,8,9,10,11,12,13], and our findings support the beneficial role of resveratrol/antioxidants in the management of age-related retinal degeneration. It should be mentioned that, although the effects of resveratrol were observed, the dose-dependent effects merit further investigation to better establish the drug effects.

The protection profiles of the rods and cones were somewhat distinguished. ONL thickness, which mainly reflects rod number/integrity, was nearly completely rescued in mice after resveratrol treatment (Figure 1A), whereas cone density and the expression levels of cone arrestin were partially rescued (Figure 1B,C). This observation suggests that other factors in addition to oxidative stress play a role in T3-induced cone degeneration. A discrepancy in the effects of resveratrol on cell death/apoptosis and ONL thickness was noted. T3-induced cell death/apoptosis evaluated by TUNEL was partially suppressed by resveratrol, whereas T3-induced reduction in ONL thickness evaluated by H&E staining was nearly completely rescued after treatment with resveratrol. At this time, it is unclear how the nearly complete rescue of retinal morphology/ONL thickness with only partial rescue of cell death was achieved. This observation suggests that oxidative stress/damage may only partially contribute to the apoptotic cell death in these retinas. It is also likely that the apoptotic cell death was not the only trigger for the reduction in ONL thickness and other types of cell death mechanisms (such as necroptosis) may have contributed to the observed reduction and responded to resveratrol. Nevertheless, the involvement of various cell death processes/mechanisms in the models studied warrants further investigations.

Notably, no functional rescue was observed in this study. T3 treatment reduced retinal function, as shown by the reduced scotopic and photopic electroretinogram (ERG) responses, similar to previous reports [17]. Concomitant treatment with resveratrol did not significantly rescue the rod and cone light responses (data not shown). The partial rescue of the cell death/cone numbers seemed insufficient for functional rescue. The resveratrol treatment conditions (dose, duration, etc.) used appeared insufficient to rescue the T3-induced deterioration of photoreceptor function, including the expression levels of phototransduction components, and to rescue the T3-induced photoreceptor degeneration to a level that is sufficient for functional recovery. We would anticipate that higher doses of resveratrol may lead to functional protection, at least in part. Further studies with adjusted experimental conditions would provide additional insight.

### 4.2. Resveratrol Preserves Photoreceptors Against Rpe65 Deficiency

Mouse models of *Rpe65* deficiency (*Rpe65^−/−^* and *Rpe65^−/−^/Nrl^−/−^* mice) display early-onset and rapid cone degeneration, and have been commonly used as models in studies of LCA [30,31,41,42]. The precise mechanism of cone degeneration in *Rpe65* deficiency is not known so far. It is generally accepted that a deficiency in visual chromophores leads to cone opsin/protein mislocalization and cellular stress. We presumed that treatment with an antioxidant might relieve cone degeneration, at least in part, in *Rpe65*-deficient mice and examined the effects of resveratrol. As anticipated, treatment with resveratrol preserved cones in these mice, rescued cone arrestin expression, and reduced cell death/apoptosis. More significantly, several genes involved in oxidative stress were upregulated in *Rpe65*-deficient mice and treatment with resveratrol significantly reduced or abolished these upregulations. To our knowledge, this is the first report showing the protection of an antioxidant/resveratrol in *Rpe65*-deficient mice. The findings support the role of oxidative stress in *Rpe65*-deficient photoreceptor degeneration and the therapeutic value of resveratrol/antioxidants in the management of LCA. It should be mentioned that, although the effects of resveratrol were observed, the dose-dependent effects merit further investigation to better establish the drug effects.

The mechanism underlying oxidative stress in *Rpe65* deficiency is yet to be determined. This could involve various factors/regulations. One of them might be associated with TH signaling, which plays a significant role in LCA cone degeneration. Inhibition of TH signaling by anti-thyroid treatment [16], targeting iodothyronine deiodinases [21,22,44], or targeting the TH receptor [23] preserved cones in *Rpe65*-deficient mice. Moreover, evaluation of the expression levels of TH signaling components in the retina, including iodothyronine deiodinases and TH receptors [21,23], suggests potential locally elevated TH signaling activity. Together with the beneficial effects of anti-TH signaling approaches, the findings from this study showing the beneficial effects of resveratrol suggest the potential contribution of TH signaling, at least in part, to oxidative stress in *Rpe65*-deficient retinas.

### 4.3. Resveratrol Reduces Retinal Stress Responses Induced by TH Signaling and Rpe65 Deficiency

Macroglial cells (Müller cells and astrocytes) are the principal glial cells in the retina that serve as sentinel/safeguard cells in response to insult/harmful stimuli. Their activation is characterized by increased expression of GFAP and formation of intermediate filaments [45]. Macroglial cell activation has been shown in a variety of animal models of retinal degeneration, including retinal detachment [46], retinal ischemia-reperfusion [47], Royal College of Surgeons rats [48], *rd* mice [49], and light-induced retinal degeneration [50,51]. The expression of GFAP, evaluated by immunolabeling on retinal sections and immunoblotting, was significantly increased in mice after T3 treatment, and treatment with resveratrol nearly completely reversed these elevations. These data are consistent with a previous report showing that treatment with NAC reversed T3-induced macroglial cell activation [17]. Our recent transcriptomic studies showed that there were approximately 180 differentially expressed genes in Müller glial cells and approximately 160 differentially expressed genes in astrocytes after T3 treatment [19]. It is likely that the activation of macroglial cells in response to T3 treatment resulted from both photoreceptor stress/degeneration and the direct action of T3 on these cells. Similarly, the observed reduction in GFAP labeling/macroglial cell activation in mice treated with resveratrol may result from both reduced photoreceptor stress/degeneration and the reduced stress responses of macroglial cells themselves per se. Similarly, *Rpe65*-deficient mice displayed profound activation of macroglial cells, and treatment with resveratrol nearly completely abolished the activation of these cells, supporting that antioxidants reduce retinal stress responses in these mice. The effect of resveratrol in reducing retinal stress responses was also supported by gene expression analysis. qRT-PCR data showed that a number of genes involved in oxidative stress responses, cell death signaling, and inflammatory responses were significantly upregulated in the retinas of mice treated with T3 and *Rpe65*-deficient mice. Treatment with resveratrol effectively inhibited or abolished these upregulations, supporting its role in mitigating retinal stress and inflammation.

### 4.4. The Benefits of Resveratrol in Animal Models of Retinal Diseases and the Potential Underlying Mechanisms

Previous studies have shown that resveratrol exerts potential benefits in animal models of retinal degeneration, including diabetic retinopathy [52,53], retinopathy of prematurity [54,55], a drug-induced rat model of retinitis pigmentosa [56], a light-induced retinal degeneration model [57], and glaucoma [28,58,59]. TH signaling has been shown to induce photoreceptor degeneration, and abnormal serum TH levels have been linked to retinal degeneration. This study demonstrates the beneficial effects of resveratrol, supporting the role of oxidative stress in TH-induced retinal degeneration. This work also demonstrates the beneficial role of resveratrol in mouse models of LCA with *Rpe65* deficiency, supporting the role of oxidative stress in disease progression. To our knowledge, this is the first study to evaluate the role of resveratrol in inherited mouse models of retinal degeneration.

The benefits of resveratrol observed in various animal models are likely mediated by its anti-inflammatory, antioxidant, and anti-angiogenic properties, which protect retinal cells from damage and reduce abnormal blood vessel growth in the retina. The present study explored the potential mechanism of resveratrol’s action by examining alterations in gene expression. We found that several genes regulating oxidative stress, cellular necroptosis signaling, and inflammation were significantly upregulated in the retinas of mice after T3 treatment and in the retinas of *Rpe65*-deficient mice, whereas treatment with resveratrol inhibited these elevations. These observations are consistent with previous findings supporting resveratrol as an antioxidant and anti-inflammatory agent in the retina. At the molecular level, resveratrol induces the NAD^+^-dependent deacetylase Sirtuin 1, which regulates cellular processes related to longevity and stress resistance [60], inhibits mitogen-activated protein kinase, and increases phosphorylation of Akt1 [54,61], leading to the reduction in cellular stress and cellular protection.

### 4.5. Limitations of the Study

This study has several limitations. First, only one dose of resveratrol was administered. This could have contributed to the observed partial rescue of retinal morphology/cell death and lack of retinal functional improvement. In a dose–response study, we may identify an optimized dose condition that allows us to see an improved rescue of the retinal phenotype. We anticipate that higher doses of resveratrol may lead, at least in part, to functional rescue. Second, the longitudinal effects of resveratrol were not evaluated in this study. In addition, the evaluation of cell death could have been more thorough and informative. Cell death, evaluated by TUNEL labeling, was only performed on retinal sections. The evaluation would have benefited from the combination of sectional labeling with whole-mount labeling for markers that are particularly critical for understanding both the retinal layers and regions. Cell death evaluation would also have benefited from co-labeling with photoreceptor-specific markers.

## 5. Conclusions

In summary, the present study demonstrated the protective role of resveratrol against retinal degeneration induced by excessive TH signaling and *Rpe65* deficiency in mice. Treatment with resveratrol preserved photoreceptors, reduced photoreceptor oxidative stress/damage, cell death/apoptosis, and upregulation of genes involved in cellular stress/death signaling and inflammatory responses, and suppressed the activation of Müller glial cells. These findings provide valuable insights into the role of oxidative stress/damage in TH-signaling-induced photoreceptor degeneration and *Rpe65* deficiency and support the therapeutic significance of resveratrol/antioxidants in retinal degeneration management.

## Figures and Tables

**Table 1:** Antibodies and other reagents used in this study.

Antibodies/Reagent	Vendor	Catalog #	Dilutions Used in IF or IB
3,3′,5-Triiodo-L-thyronine	Sigma-Aldrich	T2877	
Resveratrol	Sigma-Aldrich	R5010	
DAPI (4,6-Diamidino-2-phenylindole)	Millipore Sigma	D9542	1:2000 (IF)
Biotinylated PNA	Vector Labs	B-1075	1:200 (IF)
anti-γH2AX (p Ser139)	Novus	NB100-2280	1:200 (IF)
anti-GFAP	DAKO	Z0334	1:500 (IF)
anti-GFAP	DAKO	Z0334	1:500 (IB)
anti-β-actin	Abcam	Ab6276	1:2000 (IB)
anti-CAR	EMD Millipore	AB15282	1:500 (IB)
HRP-anti-rabbit	SeraCare	5220-0336	1:10,000 (IB)
HRP-anti-mouse	SeraCare	5220-0341	1:10,000 (IB)
Alexa Fluor^®^ 555 goat anti-rabbit IgG	ThermoFisher Scientific	A21428	1:500 (IF)
Streptavidin-Cy3	ThermoFisher Scientific	SA1010	1:500 (IF)

**Table 2:** Primers used in this study.

Gene	Accession Number	Forward Primer	Reverse Primer
*Hprt1*	NM_013556	GCAAACTTTGCTTTCCCTGGTT	CAAGGGCATATCCAACAACA
*Ripk1*	NM_009068	GGAAGGATAATCGTGGAGGC	AAGGAAGCCACACCAAGATC
*Ripk3*	NM_019955	TCTTTACTGAGACTCCCGGT	AGTTCCCAATCTGCACTTCAG
*Tnf1α*	NM_013693	CTTCTGTCTACTGAACTTCGGG	CAGGCTTGTCACTCGAATTTTG
*Tnfrsf1a*	NM_011609	CTCTGCTCTACGAATCACTCTG	CACAGCATACAGAATCGCAAG
*Tnfrsf9*	NM_011612	CCTGTGATAACTGTCAGCCTG	TCTTGAACCTGAAATAGCCTGC
*Gss*	NM_008180	GATCCTGTCCAATAACCCCAG	GCACGCTGGTCAAATATGTTC
*Ctsb*	NM_007798	AGACCTGCTTACTTGCTGTG	GGAGGGATGGTGTATGGTAAG
*Ehd2*	NM_153068	AGCTCAACGACCTAGTGAAAC	TCGCAAAGATGACAGGCAG
*Gpx4*	NM_008162	GCAATGAGGCAAAACTGACG	CTTGATTACTTCCTGGCTCCTG
*Nox4*	NM_015760	TCCAAGCTCATTTCCCACAG	CGGAGTTCCATTACATCAGAGG
*Ucp2*	NM_011671	GCATTGGCCTCTACGACTC	AAGCGGACCTTTACCACATC
*Ncf1*	NM_010876	TCATCCTTCAGACCTATCGGG	ACCTCGCTTTGTCTTCATCTG
*Nlrp3*	NM_145827	CTCCAACCATTCTCTGACCAG	ACAGATTGAAGTAAGGCCGG
*Il1α*	NM_010554	TGCAGTCCATAACCCATGATC	ACAAACTTCTGCCTGACGAG
*Il1β*	NM_008361	ACGGACCCCAAAAGATGAAG	TTCTCCACAGCCACAATGAG
*Il6*	NM_031168	CAAAGCCAGAGTCCTTCAGAG	GTCCTTAGCCACTCCTTCTG
*Dio3*	NM_172119	GTGGTCGGAGAAGGTGAAG	TGCACAAGAAATCTAAAAGCCAG
*Casp3*	NM_009810	GACTGATGAGGAGATGGCTTG	TGCAAAGGGACTGGATGAAC
*Casp7*	NM_007611	CCCACTTATCTGTACCGCATG	GGTTTTGGAAGCACTTGAAGAG
*Casp8*	NM_009812	AACTTCCTAGACTGCAACCG	TCTCAATTCCAACTCGCTCAC

## Data Availability

The data generated and analyzed during the current study are available from the corresponding author on reasonable request.
